# Single-cell transcriptome profiling of the vaginal epithelium reveals the heterogeneity of suprabasal cells

**DOI:** 10.1093/pcmedi/pbad006

**Published:** 2023-03-20

**Authors:** Chen Liang, Zixian Zhao, Sarah Liu, Ting Zhang, Wei Zuo

**Affiliations:** Shanghai East Hospital, Tongji University School of Medicine, Shanghai 200120,China; Shanghai East Hospital, Tongji University School of Medicine, Shanghai 200120,China; Shanghai East Hospital, Tongji University School of Medicine, Shanghai 200120,China; Super Organ R&D Translational Base, National MOE Engineering Research Center, General Manager's Office, Shanghai 201203, China; Shanghai East Hospital, Tongji University School of Medicine, Shanghai 200120,China; Super Organ R&D Translational Base, National MOE Engineering Research Center, General Manager's Office, Shanghai 201203, China

Dear Editor,

The integrity of the vaginal epithelium is crucial for women's reproductive health and for providing protection against HIV and sexually transmitted infections.^1^ The vagina is a tubular tract made of fibromuscular and elastic tissue that connects the cervix to the outer genitals. Its main function is to discharge uterine secretions.^2^ The vaginal epithelium (VE) is a keratinized, stratified squamous epithelium consisting of three layers: the basal layer, the suprabasal layer, and the apical cornified layer.^3^ Estrogens induce the proliferation of basal epithelial cells in the vagina. The suprabasal cells, which are no longer mitogenic, differentiate as they move up through the epithelium. The apical cells undergo keratinization, lose their nuclei and cytoplasm, and eventually shed from the surface.^4^ With multiple sexually transmitted diseases posing a significant threat to human health,^5^ it is increasingly important to understand how the vaginal epithelium regenerates to maintain homeostasis and how it differs from the neighboring cervical epithelium.

In this study, we extracted vaginal tissue from five adult virgin mice and conducted single-cell RNA sequencing (scRNA-seq) on the vaginal epithelium. We obtained a total of 7823 cells and isolated and sequenced 6187 cells from the combined duplicates that passed QC filtering. Using Principal Component Analysis (PCA) and Uniform Manifold Approximation and Projection (UMAP) projection, we clustered the cells into six distinct clusters (Fig. 1A). To identify each cell cluster, we performed differential gene expression analysis to generate cluster-specific marker genes. Known cell type-specific markers were then used to identify each cell cluster, such as Ngfr for basal cells, Mki67 and Birc5 for proliferating cells, Sprr1b and Krt1 for squamous epithelial cells, Krt8 for columnar cells, and two suprabasal clusters: a Fabp5-high suprabasal population and an Ifitm3-high suprabasal population. Additionally, to better understand the vaginal epithelium cell cycle, we performed cell cycle analysis on individual cells (Fig. 1B). Our results revealed that almost all proliferating cells and approximately half of the basal cells were in the G2/M or S phase of the cell cycle, indicating that both cell types are highly active in the mouse vagina.

**Figure 1.**
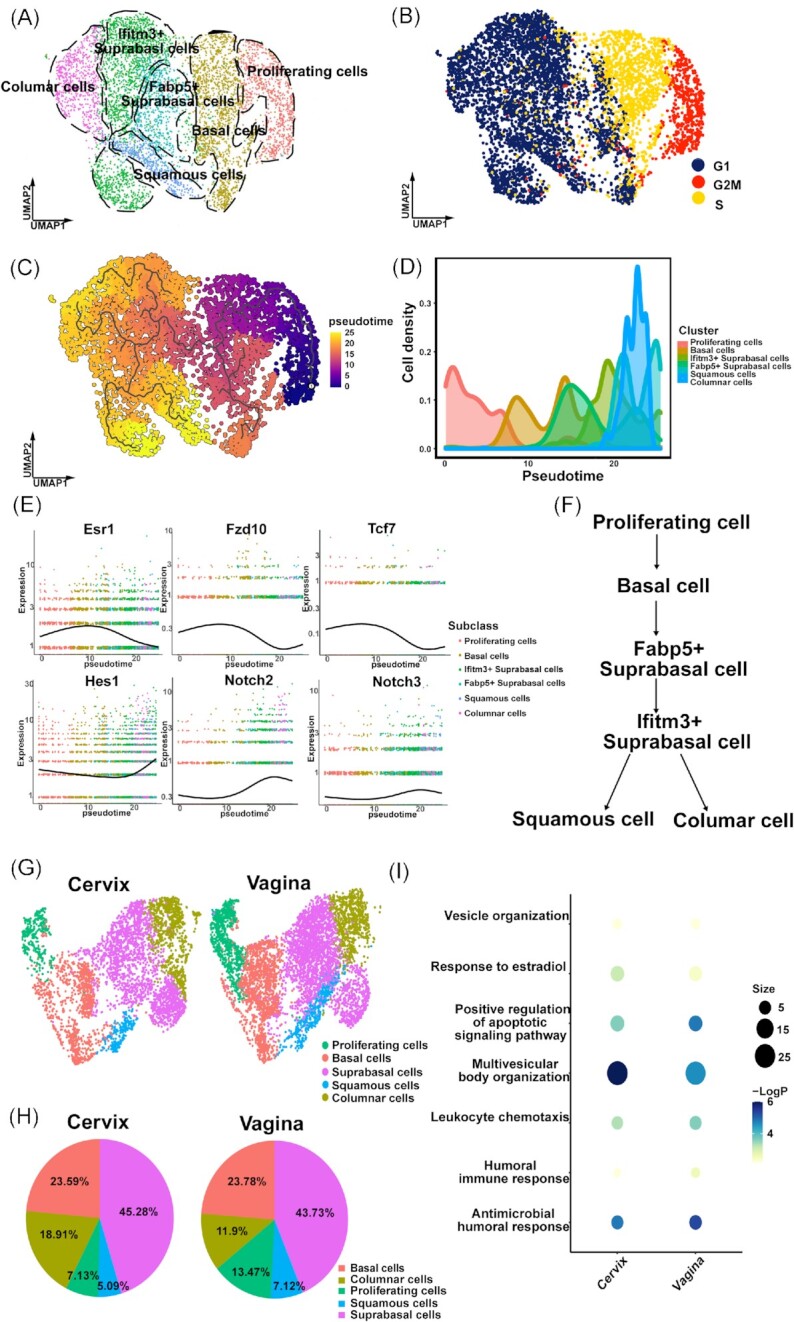
Identification of distinct cell types of the mouse vagina epithelium by single-cell RNA sequencing and integrated analysis on cervix and vagina. **(A)** Six distinct cell clusters from 10x Genomics scRNA-seq analysis visualized by UMAP. Each dot represents a single cell. Colors and numbers indicate clusters, and cell-type names are indicated with their corresponding clusters. **(B)** UMAP plot showing the cell cycle status of each cell. **(C)** Pseudo-time trajectory projected onto a UMAP of vaginal epithelial cells. Pseudo-time values are color-coded. **(D)** The cell density of vaginal epithelial cell clusters inferred cellular trajectory reflecting cervical epithelial cell differentiation. Cells are ordered by pseudo-time as computed by reversed graph embedding approach of Monocle3. **(E)** Wnt and Notch signaling pathways play a role in vaginal epithelium differentiation. **(F)** The putative differentiation paths from proliferating cells to columnar cells or squamous cells predicted by the pseudo-time analysis. **(G)** UMAP plots of cells of cervix and vagina samples, respectively. **(H)** The percentages of distinct cell clusters in the cervix and vagina samples. **(I)** Representative GO terms and pathways enriched in cluster-specific marker genes based on functional enrichment analysis.

Our scRNA-seq data revealed the existence of two distinct types of vaginal suprabasal cells. To investigate their correlations and the continuity of differentiation in the vagina, we utilized the reversed graph embedding method to computationally infer the differentiation trajectory of vagina epithelial cells from the scRNA-seq atlas through pseudo-time mapping (Fig. 1C). Our analysis revealed that while both Fabp5-high suprabasal cells and Ifitm3-high suprabasal cells are suprabasal cells, the Ifitm3-high suprabasal cells are likely to be differentiated from the Fabp5-high suprabasal cells (Fig. 1D).

Our next objective was to identify signaling pathways that regulate the differentiation of basal and suprabasal cell lineages. Through our pseudo-time analysis, we found that several Wnt signaling genes (*Esr1, Fzd10*, and *Tcf7*) were highly expressed in basal cells but started to decrease in Fabp5-high suprabasal cells. In contrast, Notch signaling genes (*Notch2, Notch3*, and *Hes1*) showed low expression levels in basal and Fabp5-high suprabasal cells but began to increase in Ifitm3-high suprabasal cells and terminally differentiated cells (Fig. 1E). Our findings suggest that the Wnt-related pathway may be involved in maintaining the self-renewal of basal cells,^6^ while the Notch-related pathway could promote cell maturation and epithelium differentiation^7^ (Fig. 1F).

We were particularly interested in exploring the similarities and differences between the vagina and the cervix, as both are stratified epithelia located in the lower female reproductive tract that originate from the Müllerian duct during development, which is formed by the proliferation of mesodermal cells.^1^ To investigate this, we performed an integrated analysis of two single-cell sequencing datasets.^8^ After accounting for batch effects, we found that the cells in both datasets exhibited similar biological states. Specifically, both the cervix and the vagina were composed of five cell types: proliferating cells, basal cells, suprabasal cells, squamous cells, and columnar cells (Fig. 1G). Notably, basal cells, which play a key role in regeneration, and suprabasal cells, which are constantly renewed, were present with similar proportions in both tissues. However, the proportion of columnar cells responsible for secretion was larger in the cervix than in the vagina, indicating the dominant secretory function of the cervix. Conversely, the proportion of proliferating cells and defensive squamous cells was larger in the vagina than in the cervix, suggesting that the vagina has more active cell renewal activities as the first line of defense against pathogenic invasion in the lower reproductive tract^9^ (Fig. 1H).

To gain further insight into the similarities and differences between the cervix and vagina, particularly regarding the two types of columnar cells, we performed Gene Ontology (GO) and pathway analyses (http://metascape.org/) to examine the biological implications of genes that were significantly up-regulated in these two cell types. Our findings indicated that both types of columnar cells were enriched in genes related to secretion, such as those involved in vesicle and multivesicular body organization, which are essential for cell secretion and transport. Additionally, as the first line of defense in the lower reproductive tract, vaginal columnar cells are primarily responsible for secreting anti-microbial substances, which are accompanied by a stronger leukocyte chemotaxis response. Vaginal columnar cells were also found to have higher levels of apoptotic signals and more prominent cell renewal compared to cervical columnar cells. Furthermore, both organs were enriched in estrogen-related signals, although the cervix exhibited slightly higher enrichment levels (Fig. 1I). These results provide insights into the similarities and differences between columnar cells in the cervix and vagina, which are evolutionarily adapted to the distinct functions of the organs.

In our study, we classified vaginal epithelial cells into six distinct clusters, including two subpopulations of suprabasal epithelial cells with a defined temporal differentiation sequence. Additionally, we uncovered several unexpected dissimilarities between cervical and vaginal epithelial cells. Our findings carry significant implications for comprehending the role of vaginal stem/progenitor cells in maintaining tissue homeostasis, regeneration, and disease progression *in vivo*. They may also aid in developing novel cell-based therapies in the future.

## Supplementary Material

pbad006_Supplemental_File
