# Supplementary material for: Single-cell transcriptome profiling of the vaginal epithelium reveals the heterogeneity of suprabasal cells
Source: Precis Clin Med. 2023 Mar 20;6(1):pbad006. doi: 10.1093/pcmedi/pbad006 (PMC10065133; doi:10.1093/pcmedi/pbad006)
Supplement: pbad006_Supplemental_File [file pbad006_supplemental_file.docx]

**Supplementary materials**

**Method**

**Mice tissues**

All animal experiments were carried out by Chinese National Guidelines GB/T 35892–20,181, as well as under the guidance of the Institutional Animal Care and Use Committee of Tongji University. Mice were kept in a temperature-controlled SPF (Specific Pathogen Free) environment with a regular light/dark cycle and provided with adequate rodent diet and water. All mouse experiments were performed on 8–10-week-old female mice and represent a minimum of n = 5 mice in all groups. All mice were randomly allocated to the experimental groups.

**Single-cell RNA sequencing analysis**

Single cells were captured and barcoded in 10x Chromium Controller (10x Genomics). Subsequently, RNA from the barcoded cells was reverse-transcribed and sequencing libraries were prepared using Chromium Single Cell 3’v2 Reagent Kit (10x Genomics) according to the manufacturer’s instructions. Sequencing libraries were loaded on an Illumina NovaSeq with 2 × 150 paired-end kits at Novogene, China. Raw sequencing reads were processed using the Cell Ranger v.3.0.0 pipeline from 10X Genomics. In brief, reads were demultiplexed and aligned to the mouse mm 10 genome and UMI counts were quantified per gene per cell to generate a gene-barcode matrix. Data were aggregated and normalized to the same sequencing depth, resulting in a combined gene-barcode matrix of all samples. Seurat v.3 was used for quality control, dimensionality reduction, and cell clustering. The low-quality cells with less than 200 or more than 6000 detected genes were removed, or their mitochondrial gene content was > 10%. Genes were filtered out that were detected in less than 3 cells. This filtering step resulted in 18,674 genes X 6187 cells. The filtered gene-barcode matrix was first normalized using the ‘LogNormalize’ methods in Seurat v.3 with default parameters. The top 2000 variable genes were then identified using the ‘vst’ method in the Seurat FindVariableFeatures function. Variables ‘nCount_RNA’ and ‘percent.mito’ were regressed out in the scaling step and PCA was performed using the top 2000 variable genes. Then UMAP (uniform manifold approximation and projection) was performed on the top 50 principal components for visualizing the cells. Meanwhile, graph-based clustering was performed on the PCA-reduced data for clustering analysis with Seurat v.3. The immune cell cluster was removed and the other cells were re-clustered using the same parameter mentioned above in the clustering step. MAST in Seurat v.3 (FindAllMarkers function) was used to perform differential gene expression analysis. For each cluster of epithelial cells, DEGs were generated relative to all of the other cells. A gene was considered significant with adjusted P < 0.05 (P values were adjusted by false discovery rate in MAST). Violin plots were performed by the function VlnPlot in Seurat with the default parameters. Expression heatmap for marker genes was performed by the function DoHeatmap in Seurat with the default parameters.

**Cell cycle analysis**

A cell cycle score was assigned to each cell according to its gene expression of G2/M and S phase markers based on the scRNA-seq data. Based on this scoring system, each cell was classified in either G2/M, S, or G1 phase using the CellCycleScoring function in Seurat. The cells at different cell cycle classifications were visualized in the UMAP map.

**Single-cell trajectory analysis**

To infer the cluster and lineage relationships between the different epithelial cell types identified in scRNA-seq data, Monocle3 was used (https://github.com/cole-trapnell-lab/monocle3). UMAP embeddings and cell clusters generated from Seurat were used as input, and trajectory graph learning and pseudo-time measurement through reversed graph embedding were performed with Monocle3. To construct a single-cell pseudo-time trajectory and to identify genes that change as the cells undergo transition, the Monocle2 (version 2.4.0) algorithm was also applied to our data. Genes for ordering cells were selected if they were expressed in ≥10 cells, their mean expression value was ≥0.1, and their dispersion empirical value was ≥2. Cells were ordered along the trajectory and their trajectory was visualized on the reduced dimensional space. Significantly changed genes along the pseudo-time were identified using the differential GeneTest function of Monocle2 with q-value < 0.01.

**Gene Ontology (GO) analysis**

GO biological process and pathway enrichment analyses of differentially expressed genes in scRNA-seq data were performed using Metascape(version 3.5) (http://metascape.org), and the results were visualized with the ggplot2 R package. GO terms with a P value less than 0.01 were considered significantly enriched by differentially expressed genes.
